# What effects of COVID-19 on regional economic resilience? Evidence from 31 provinces in China

**DOI:** 10.3389/fpubh.2022.973107

**Published:** 2022-07-28

**Authors:** Tian Meng, Congying Tian, Henglong Zhang, Chun Kwong Koo

**Affiliations:** ^1^School of Economics, Shanghai University, Shanghai, China; ^2^Xi'an Jiaotong-Liverpool University, Suzhou, China

**Keywords:** COVID-19, regional economic resilience, global financial crisis (CFC), counterfactual method, geographic detector model

## Abstract

After the 2008 global financial crisis, more and more scholars began to focus on economic resilience. In 2020, the outbreak of COVID-19 made the public aware of the importance of regional economies to resist and adapt to external shocks. Based on cross-sectional data from 2020 and 2021, this paper uses causal inference counterfactual to assess the regional economic resilience of various Chinese provinces under the COVID-19 pandemic, and analyzes the determinants of regional economic resilience through geographic detector models. It is found that (1) from the regional perspective, the eastern and central regions are the first to be affected by the epidemic, and their economic resistance is lower than the national average, but the eastern and central regions can stabilize the development trend of the epidemic earlier; (2) from the perspective of provinces, developed provinces are more vulnerable to the epidemic in the early stages than backward provinces; (3) government forces and social environment play an important role in regional economic resistance and adaptation in the initial stage of epidemic impact. Therefore, at the critical moment of China's post-epidemic economic recovery, it should be noted that the regional response to the epidemic depends on the path of action and the specific environment, and cannot be “one-size-fits-all.” Pay close attention to the key role of government and the management of risk prevention. The region has established sound public health policies, systems and mechanisms.

## Introduction

COVID-19 is spreading globally, with outbreaks occurring in more than 200 countries and territories, showing the characteristics of a pandemic. The spread of the epidemic has posed a serious threat to the world's public health environment and people's lives and health safety, and has caused a huge impact on social and economic development. It seems that over the past 20 years, countries and regions have been increasingly exposed to uncertain risks and shocks. In particular, the financial crisis in 2008 attracted great attention from the academic community on how the region responded to special events and uncertain shocks and how to recover (1, 2). Since then, regional resilience studies have followed suit. Based on the theories of neoclassical economics and evolutionary economic geography, studies on regional resilience are developing rapidly. The concept of resilience has been widely applied in geography, disaster science, ecology and economics with the idea of “bottoming out” or recovery equilibrium (3). However, in the economic geography, regional economy to recover from the impact resilience is considered to be regional success and the ability to get rid of the existing or potential growth path, namely the region after the impact, resist force to keep the economic system, the structure and functions of the original, but still needs to be adaptive change the structure and function make it faster recover from recession (4). Therefore, regional economic resilience varies with time, which depends on the characteristics of shocks and the structure and nature of regional economy. Subsequently, economic geographers try to de-contextialize regional economic resilience and put it into a broader field, pointing out that regional economic resilience is not only the ability to deal with a shock, but also the long-term ability to adapt to the ubiquitous uncertain risks (4–6). Resilience is essentially a central feature of the evolution of economic systems in an uncertain environment: an endless process in which actors and actors prepare for regional sustainable growth and adapt to natural and man-made challenges. For economic geographers, the main research theme is to explain why regional economies are different in their resilience to different shocks, and what determines these different impacts over time and space (4, 7, 8).

Thus, this paper can identify three main areas of concern. First, resilience is increasingly recognized as a multidimensional, complex system process that not only resists and adapts to shocks, but also has the long-term capacity to expand into new paths of regional development. A revised conceptual framework of systemic resilience includes four aspects: vulnerability, resilience, robustness and resilience. It explains the various possible responses of regional economies to severe economic downturns and proposes that the characteristics of shocks and their own economic structure have an important impact on the response process (6). Secondly, both quantitative and qualitative studies show that resilience is not only dependent on the way it responds to shocks, but also depends more on the economic foundation and artificial control ability of the system itself. In other words, resilience can be changed by both the historical succession structure and the institutional structure of an area (9–11). Finally, the type of shock itself is an important starting point for understanding the resilience of regional economies. From a spatial perspective, global-to-regional shocks are mostly sudden, large-scale, and uncertain events, such as the great Depression caused by the global financial crisis in 2008 and the global spread of COVID-19 in 2020. Moreover, even one-off emergencies can have lasting or permanent effects (12). For instance, In other parts of the regional industry will continue to accumulate in the competition, if there is no more competitive over time or region industry to be eliminated, this may be due to the district sunk costs and inherited systems and mechanisms, the rigidity of regional development path and hinder the transformation, declining market share, business failures, industry downturn, labor loss, even the economy is flagging. Regional economic decline may be slow over time, but it becomes a sudden and urgent shock when it reaches a tipping point or is pushed by external forces. Therefore, factors affecting regional economic resilience can develop slowly and gradually, but resilience should be the actual response of the region to shocks, so as to clearly measure regional resilience and the evolution of development paths (7, 13).

Since the outbreak of the novel pneumonia outbreak, confirmed cases have been reported in more than 100 countries and regions around the world. As a continuing global epidemic, it has led to a true global public health crisis. According to WHO statistics, as of April 20, 2022, the number of confirmed COVID-19 cases in the world had exceeded 504.4 million and the number of related deaths had exceeded 6.2 million, with the United States, Brazil and India among the most affected countries. The global economy has suffered a severe setback due to the sudden outbreak of the epidemic. The economy has declined, consumption has contracted and the number of unemployed has soared. The spread of the epidemic has had a major impact on global trade and China's import and export trade. Arguably, in just a few months, the pandemic has caused the greatest global economic disruption since the Great Depression (14). Given the unprecedented scale and depth of the shock, the concept of resilience is again in focus.

More and more scholars have begun to focus on the geographically uneven impact and consequences of the epidemic. In particular, different countries have significant differences in the spatial nature of communication, the vulnerability of human life, the quality of health care and the effectiveness of national policies (15). Other scholars believe that COVID-19 itself reflects more power imbalance in space, as well as social and political contradictions between countries and regions (16). This requires consideration of the collective characteristics and dynamic capabilities of a region supported by multiple scalar interactions between economic structure, government institutions and social environment (17, 18). In this respect, the positioning of this study differs from the recent COVID-19 centric literature, which focuses on the socio-economic effects of novel Coronavirus (14); Novel Coronavirus socio-economic Impacts, policy responses and opportunities (19); policy and academic insights on the economic consequences of COVID-19 (20); evolution and patterns of spatial spread of COVID-19 (21); 10 measures in public health management (22); the effectiveness and relevance of different responses to the pandemic (23); the relationship between public health crisis capacity and national mortality rates in novel Coronavirus (24); the impact of economic policy uncertainty on export trade under COVID-19 (25); the impact of industrial and government institutions on economic resilience under COVID-19 (17). Although the existing literature has studied the impact of COVID-19 qualitatively and quantitatively, it is surprising that the topic of how regional economies can resist and adapt to shocks has not yet dominated the literature on COVID-19 (26). Therefore, the purpose of this study is to fill in the gap of relevant research according to the above-mentioned discussion objectives.

It is worth highlighting that the existing literature includes: (a) differences in economic resilience under external shocks (not only COVID-19, but also the wenchuan earthquake in China and the subprime crisis in the us) and its influencing factors (21, 27, 28) and (b) seek to find the relationship between regulatory measures taken by government agencies to respond to COVID-19 and regional economic resilience (17, 18). A common weakness of the underlying literature is its failure to compare and assess regional resilience and adaptation to the impact of COVID-19 and the delayed role of government forces in the economic system, with a particular emphasis on regions in China. In order to solve the accompanying gap in the literature, the marginal contribution of this paper has two aspects: first, it compares and evaluates the economic resilience of various provinces in China, and restores their spatial distribution and changes in confirmed cases. Second, it examines the impact of government forces and social environment on regional economic resilience under COVID-19, adding new perspectives and insights to the research on regional economic resilience.

The rest of the paper is arranged as follows. Section Literature review and hypotheses reviews the literature on regional economic resilience and the 2008 financial crisis, and makes assumptions about the factors that COVID-19 may affect regional resilience. Section Materials and methods introduces the study area, methods and data. Section Comparison between the two periods: differentiated resistance to COVID-19 shows resilience and spatial distribution of confirmed cases. Then, Section Determinants of the differential resistance to COVID-19 discusses the determinants of China's regional economic resilience imbalance under the COVID-19 pandemic. Section Conclusion and enlightenment conclusion and enlightenment.

## Literature review and hypotheses

### Regional economic resilience and 2008 financial crisis

The word “resilio” comes from Aladdin's “Resilio,” which originated from physics and is defined as the ability of a system to maintain and restore to the original state after external impact. Early studies on resilience focused on equilibrium theory, including engineering resilience and ecological resilience. Holling (29) first introduced resilience into ecology and defined it as “engineering resilience,” describing the ability of an ecosystem to recover to its initial state after an impact. Later, this concept was revised and supplemented, and the idea of “ecological resilience” emerged, which means that when a system encounters an impact or damage, it can not only recover to its initial state, but also generate resistance that enables it to find a new equilibrium state (30). In other words, the system should not include only the unique equilibrium state emphasized by engineering resilience (31). With the continuous improvement of resilience theory, the concept of adaptive resilience from the perspective of evolutionary theory was proposed. In the 1990s, Gunderson (32) proposed the adaptive cycle theory, and combined the connotation of “engineering resilience” and “ecological resilience,” gradually evolved the “adaptive resilience.” According to adaptive resilience theory, resilience is an inherent attribute of economic system and exists independently of external disturbance. Adaptive resilience emphasizes the dynamic adjustment ability of the economy itself by gradually adapting to the external environment, which lays a good foundation for the concept of resilience to enter the field of economics. It is generally believed that Reggiani et al. (33) introduced the concept of resilience into economics for the first time. He defined economic resilience as the ability of economic system to maintain and restore its structure to a stable state after being impacted by external uncertainties in the process of spatial dynamics. Economic resilience includes not only the ability of the economic system to withstand external shocks, but also the ability to capture transforming external opportunities (34). Specifically, economic resilience involves different levels such as households, firms and markets, and is an inherent response mechanism of an economy, namely the ability to cope with external shocks to avoid losses (35). Of course, economic resilience is not to achieve equilibrium, but to gradually evolve into a complex adaptive system through continuous absorption of external environmental information (36). Subsequently, scholars began to introduce the idea of resilience into regional economics, opening up a new research field of regional economic resilience. Foster (37) introduced the concept of regional resilience for the first time, he pointed out that regional economic resilience refers to the ability of a region to recover and resist disturbances when the external environment is violently turbulent. Although regional economic resilience can be regarded as the ability of the economic system to self-recover after shocks, such recovery often deviates from the initial state (1). This bias may be due to resilience's lag, as economies decline, regional economic resilience is a dynamic process with four stages including resistance, recovery, re-orientation and renewal (4). Martin and Sunley (6) further extend the more normative concept of resilience by introducing the concepts of vulnerability, resistance, robustness and recoverability. Vulnerability refers to the sensitivity or tendency of a region's economy to the structure of regional growth before the impact. Resistance refers to the degree of direct response to shocks, which is related not only to the nature of shocks, but also to the properties of regional economic systems (5). Robustness and resilience represent adaptive sectors that associate actions and decisions with shocks and recoveries during economic downturns (38).

The 2008 financial crisis swept the world, which was the most serious economic recession in major developed countries in history, and also slowed down the economic growth of many emerging economies such as China (3, 11). At the same time, the international economic environment is changing, the impact of external uncertainties is rising, China has entered a “new normal” of shifting growth drivers, and unbalanced development among regions is becoming more prominent. The occurrence of global financial crisis makes the regional response to special events and uncertainty shocks more and more attention. The development of a country or region is not a smooth and gradual process, and will be affected by various internal and external shocks. In the process of coping with these shocks, the development path of the region may change, and different shocks will also cause differences in the form and reflection mechanism of regional economic resilience. Meanwhile, the economic resilience of different regions in different national backgrounds shows a heterogeneous pattern. A complex set of influencing factors, which can be economic, social, institutional or structural, shape the nature of regional resilience at local and peripheral scales (38). However, it is not clear what determines resilience (7).

### Hypotheses: What factors affecting regional resilience under COVID-19?

The financial crisis of 2008 and the COVID-19 pandemic are bound to differ, at least in their goals, severity, scope and duration, but they both had a huge impact on the economy. Three key differences in economic resilience between the two crises can be identified. First, while the main issue of the financial crisis is the recovery of the financial system and economic growth on the demand side, COVID-19 is a public health crisis that affects almost all types of social activities globally (26). The first response associated with resilience is to save lives and contain the spread of the virus. In this regard, government-led containment measure—medical assistance, personnel and supplies, home-based school attendance, and transportation restrictions—are indeed leading forces that directly affect the regional economy. Second, traditional regional economic structures have lost their resilience in the face of COVID-19. For example, there is evidence that economic performance is more vulnerable in areas where Labor and transport are intensive or where there is more international trade in the supply chain (39). This indicates a lack of domestic safety awareness in existing global supply chains (40). Finally, while businesses were important players in the resilience of the 2008 financial crisis, the COVID-19 pandemic is a different type of major player. Regional economies under COVID-19 are sensitive to the role of state institutions and governments in containing the pandemic (which may have negative effects) and in the subsequent restoration of socio-economic order (23).

Based on the above discussion, this paper hypothesizes that both social environment and government governance influence regional economic resilience in the face of COVID-19. A further review of the literature on COVID-19 suggests four major hypotheses that could affect regional economic resilience in the context of COVID-19 shock.

#### Economic openness

In the existing literature, the region with high degree of economic openness usually refers to the developed economy and trade dependence. They can attract a large number of foreign companies, capital and technology, and more effectively mobilize social capital to promote regional development, thus improving the ability to cope with external shocks (6, 41). However, the validity of this theory largely depends on the nature and scope of external shocks. COVID-19 is a global external shock that has led to massive work stoppages, port closures and the suspension of international trade in major economies around the world, including China, the US and Europe. Regional economies that are more closely linked to the rest of the world are likely to exhibit greater vulnerability and risk in COVID-19 (14). At the same time, import and export trade is restricted globally, and regions that rely more heavily on trade face greater economic difficulties and pressures (38). In this regard, it can be assumed:

H1. Regions with more open economies are more vulnerable to COVID-19 and have weaker economic resilience.

#### Government power

Government intervention, policy environment and economic development strategy can all have a great impact on regional economic resilience. Many scholars believe that when confronted with external shocks such as financial crisis, natural disaster and public health emergency, the government can quickly allocate social resources to successfully overcome the crisis. The nature of the crisis (for example, its origin, duration, scope and impact) will play a key role in underpinning the way government actions respond to the crisis (12). Unlike the 2008 financial crisis, which mainly affected specific industries, COVID-19 is a global pandemic and the government is responsible for saving lives and containing the virus. For example, after the outbreak of COVID-19, local governments in China have taken a series of measures, including medical support, material transportation and public safety, to fight the spread of infectious diseases. These measures inevitably lead to economic stagnation. As Swanstrom (42) found in his research, the impact of government power on economic resilience is two-sided, with a people-oriented government system impeding the development of economic and social activities when emergency measures are taken.However, this stagnation is temporary, especially as the epidemic has been gradually brought under control in China since 2020. In this sense, restoring the pre-pandemic government dispatch will help restore the original economic and social activities. Therefore, it can be assumed:

H2. The foundational strength of the government's response to COVID-19 has an important impact on the resilience of regional economies, but may have a negative impact.

#### Social environment

In the early 20th century, with the gradual deepening of economic network connection, relevant scholars found that the development of regional infrastructure could effectively drive the development of local economy, and the level of infrastructure development was closely related to the level of economic development. For example, in areas with a relatively complete urbanization degree, attracting the inflow of foreign population drives the local demand for goods and services, which leads to the expansion of domestic demand and the promotion of regional economy (43). However, there is heterogeneity in regional economic development, especially in the face of external shocks or favorable policy news. Only areas with strong economic resilience and a sound foundation will take the lead in achieving economic growth, radiating to the surrounding areas. In terms of the research on the impact of transport factors on economic resilience, only some articles put forward the role of transport infrastructure construction in promoting economic resilience from a qualitative level, and did not consider the role of external shocks. In addition, since the existing research knowledge on the influencing factors of regional resilience mainly focuses on the industrial structure and government intervention of the economic system itself, exploring the impact of social environment on China's regional economic resilience may provide some new insights into the literature. Therefore, it can be assumed:

H3. Social environment has a positive impact on regional economic resilience. Areas with better infrastructure are more resistant.

#### Innovation ability

Regional innovation capacity has gradually become an important factor to promote the sustainable development of regional economy. It is generally believed that the stronger the regional innovation capacity is, the stronger the economic resilience will be. This can be proved by the transformation cases of New Orleans, Cape Town and Phoenix, in which innovation is the driving factor of regional transformation and upgrading, and sustainable development can be achieved through the transformation of system structure and function (44). Meanwhile, taking Spain's service industry as an example, for every unit of innovation input, the local economic resilience value can increase by 0.12 units (45). On the other hand, in relatively conservative areas, people tend to have stable jobs and lack of innovation spirit, while in areas with high degree of openness, people are more likely to have entrepreneurial spirit and enhanced innovation ability, which is conducive to the benign development of regional economy (46). In addition, Doran and Fingleton (47) analyzed the economic resilience of individual employment in Europe in response to the 2008 economic crisis, and found that regions with higher educational level were more resilient than those with lower educational level. Therefore, it can be assumed:

H4. Regional innovation capability has a positive impact on regional economic resilience. The higher the level of innovation in a region, the stronger its resistance.

## Materials and methods

### Study area and data

The COVID-19 pandemic poses unprecedented challenges to human health, the world economy and the global industrial chain and value chain. China was the first country to be caught in the epidemic, and the fastest to stabilize the epidemic and take regular measures to resume work and production. However, there are significant regional differences in response to and control of the epidemic. In this regard, exploring the regional impact of COVID-19 in China can lead us to further study the complexity of regional economic resilience. On the other hand, many studies have highlighted the heterogeneity of resilience between regions, mainly due to regional differences in social environments and resource allocation. However, the role of government agents and social foundations in shaping regional resilience is rarely studied in the existing literature. Arguably, the body of government and its reserve capacity to deal with the spread of a pandemic, as well as the social base accumulated in the past, are critical to resilience.

Based on this, the paper took 31 provinces and cities in mainland China as research units to explore the impact of COVID-19 on regional resilience. Given the limited data available at the time of writing, our most recent data can only go back to 2021. More specifically, this paper used data from 2019 to 2021 to measure the biennial regional Resistance Index, which is derived from the “China Statistical Yearbook.” The map vector data was obtained from the Earth System Science Institute's data sharing platform (http://www.geodata.cn).

### Measurement methods

There is no single consistent way to analyze regional resilience to economic cycles, which are constructed for recessions and subsequent recoveries (8). Due to the different nature of shocks, different study times and different data sources, regional economic resilience is measured in different ways. Martin (4) provides a useful and simple analytical framework. He believes that regional economic resilience can be identified by four characteristics, namely vulnerability, resistance, robustness and recoverability. While vulnerability and resistance are often determined by the inherent and inherited assets and structural attributes of the region exposed to shocks, robustness and resilience refer to the role of the economic system in deliberately responding to shocks in order to recover and adapt to shocks (8). Putting them in the context of COVID-19, regional economic resilience is a region's capacity—the capacity of its socio-economic systems, resource allocation, institutional arrangements, etc., to contain the spread of the virus and save lives in the short term, and to enhance resilience and regional economic recovery in the long term. As the epidemic was not fully over at the time of writing, economic resilience in this paper mainly relates to vulnerability and resistance, although some areas are resilient. Therefore, this paper will focus on measuring the resistance indicators of regional economic resilience.

Existing literature has proposed several methods to measure the speed and magnitude of impact response in a region, such as descriptive case analysis, statistical analysis, time series of impulse response, and counterfactual methods of causal inference to measure regional resistance and resilience. Case analysis, statistical analysis and causal inference are widely used in existing studies. It is difficult to quantify the differences and influencing factors of regional economic development paths by qualitative case analysis alone. Statistical analysis and causal inference counterfactual method, data is easier to obtain, analysis is more extensive and can be compared dynamically; The empirical study of time series of impulse response is more rigorous in science, but requires higher data, so it is difficult to obtain or use data in a long time period in general studies. Based on this, this paper gives priority to the counterfactual method of statistical analysis and causal inference. However, in the process of estimating resistance, the rate of change of output in statistical analysis is positive and negative, and it is impossible to make a comparison after directly calculating the sensitivity index. Sensitivity index is calculated again after dimensionless processing of output growth rate. The natural discontinuity grading of this result is difficult to capture the discrete distribution of sectional data, and is inconsistent with the confirmed cases in most provinces. Based on this, this paper draws on the counterfactual method of causal inference by Martin and Sunley (6) and Doran and Fingelton (48) to compare the actual and expected changes of regional economic output and calculate the resistance of provinces to shocks. The formula for calculating the change in regional expected economic output is as follows:


(1) $$\begin{array}{r} {\left(\Delta R_{i}^{t+k}\right)}^{expected}=\overset{n}{\underset{j}{\sum }}R_{ij}^{t}∙G_{n}^{t+k} \end{array}$$

Where ${\left(\Delta R_{i}^{t+k}\right)}^{expected}$ represents the expected change of output value in region *i* in period *t*+*k*, $R_{ij}^{t}$ is the output value of industry *j* in region *i* at initial time *t*, and $G_{n}^{t+k}$ is the change ratio of national output value in period *t*+*k*. Then, the measurement of regional resistance can be expressed as:


(2) $$\begin{array}{r} Resis_{i}=\frac{\left(\Delta R_{i}^{contraction}\right)-{\left(\Delta R_{i}^{contraction}\right)}^{expected}}{\left|{\left(\Delta R_{i}^{contraction}\right)}^{expected}\right|} \end{array}$$

Where $\left(\Delta R_{i}^{contraction}\right)$ represents the actual change value of output in region *i* in period *t*+*k*. According to the formula, the central value of resistance is 0. When the resistance is positive, the region is better able to withstand the impact of COVID-19 than the national average, and the regional economy is more resilient, and vice versa.

### Geographical detector model

This paper developed a geodetector model to analyze the factors influencing regional resilience. Geographic detectors were first applied to the study of epidemic and geographic-related risk factors, and were later widely used to identify different socio-economic factors and their interactions (49). Compared with traditional statistical methods, this model involves fewer assumptions and can be more convenient for processing mixed data of different types. In addition, geographic detectors identify the correlation between variables by observing their spatial distribution (50). If there is a significant spatial consistency between a factor and regional resilience, it is considered that the factor plays a decisive role in regional resilience. It analyzes the explanatory power of factors related to the explained variables one by one, so the explanatory power of a particular factor is not affected by other variables. Therefore, this method is suitable for studying the factors influencing regional economic resilience under the COVID-19 shock.

Assumed that the resistance of each province is *U*, the number of provinces is *n*, and the influencing factors on resilience are *D* = {*D*_*i*_} (*i* represents the classification number), and the total is *m*. Overlay *U* and *D*, as well as the discrete variance of *U* in the subregion of the influence factor, are defined as $\sigma_{U_{D,i}}^{2}\left(i=1,2,\ldots m\right)$. Therefore, the determinants *D* = {*D*_*i*_} on regional economic resistance can be expressed as:


(3) $$\begin{array}{r} P_{D,U}=1-\frac{1}{n\sigma_{U}^{2}}\sum_{i=1}^{m}\left(n_{D,i}∙\sigma_{U_{D,i}}^{2}\right) \end{array}$$

Where *P*_*D, U*_ is the explanatory power of the impact factor *D*_*i*_, *U* is the regional economic resilience, *n*_*D, i*_ is the number of provinces in the sub-region with the impact factor. *P*_*D, U*_∈[0, 1]. When *P*_*D, U*_ = 0, it indicates that regional economic resilience is randomly distributed. The larger *P*_*D, U*_ is, the greater the influence of various factors on resilience.

### Index composition and data description

Based on our assumptions and the characteristics of the COVID-19 pandemic, this paper focuses on four major factors affecting the resilience of China's regional economies, including nine indicators. They are regional economic strength (economic openness and economic level), government power (medical and health level, grain and oil reserves, public safety), social environment (urbanization, transportation infrastructure) and innovation ability (science and technology level, educationa level). All definitions and descriptive statistics for these variables are shown in Table 1.

**Table 1:** Variables and descriptive analysis.

**Variables**	**Definition**	**Min**	**Max**	**Mean**
Regional economic resilience	The index of resistance	2.946	3.401	0.167
Economic openness	The proportion of total import and export trade in GDP	0.008	0.891	0.224
Economic level	Real GDP per capita	19,653	119,711	46,834
Medical and health level	Share of health expenditure in government budget	0.056	0.121	0.088
Grain and oil reserves	Share of reserves expenditure in government budget	0.002	0.012	0.004
Public safety	Share of public safety expenditure in government budget	0.040	0.086	0.055
Urbanization	Ratio of urban population to total population at year-end	0.358	0.893	0.627
Transportation infrastructure	Ratio of railway and road mileage to area	1.44	188.29	45.62
Science and technology level	Share of technology expenditure in government budget	0.004	0.058	0.023
Education level	Average years of schooling	5.827	11.135	8.709

China Statistical Yearbook, Statistical Yearbook of Chinese Provinces and Seventh National Census data.

First, regional economic strength describes the overall level of economic development of a region. In this paper, economic openness and real per capita GDP are used as surrogate indicators to measure regional economic strength. Among them, the degree of economic openness of a region can be measured by the ratio of total import and export trade to GDP. In addition, the real per capita GDP index can truly reflect the changes in the real living standards of people in a region, and can better reflect the economic strength of a region than GDP.

Second, government power, which reflects the role of government institutions in resisting and responding to crises. Health spending, grain and oil reserves reflect the efforts of government agencies to cushion the regional economy against the impact of COVID-19. These variables also include the life and health of the majority of people, as well as the safety of public and private property, namely the variable public safety, which is also the protection and protection the legitimate rights and interests of citizens by government departments.

Third, the social environment, which takes into account the region's urbanization rate and transport infrastructure construction, represents the inherited impact of a region's social organization and governance on regional resilience. The urbanization is measured by the current widely accepted statistical yardstick, that is the ratio of urban population to regional total population at the end of each province. In addition, for transportation infrastructure, the density of transportation network, namely the ratio of the sum of railway and highway mileage to the area of the province, was selected as a proxy variable.

Fourth, innovation ability, which describes the ability of a region or social organization to continuously provide new ideas, new theories, new methods and new inventions in various fields of practical activities. It generally includes two parts: one is the innovation of science and technology, which takes the share of science and technology expenditure in the government's general budget expenditure as a surrogate indicator; the other is the innovation of talents, which is expressed by the average number of years of education in the region.

## Comparison between the two periods: Differentiated resistance to COVID-19

**Figure 1:**
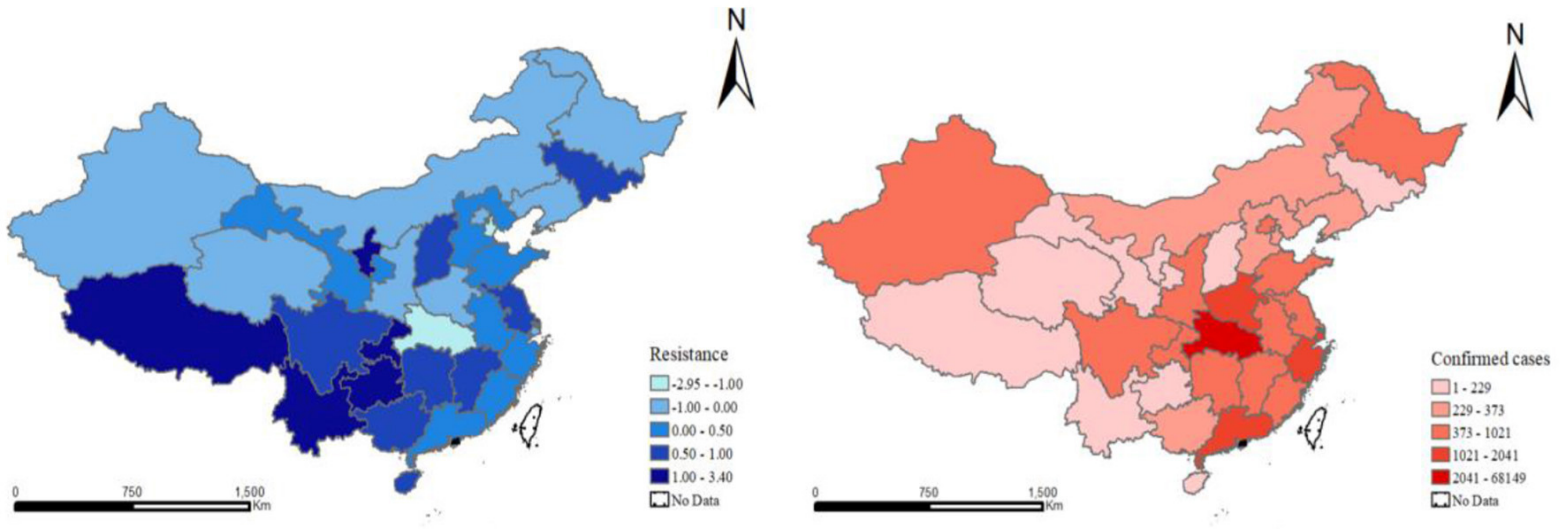
Regional economic resistance and geographic distribution of confirmed cases in provinces of China during COVID-19 in 2020.

**Figure 2:**
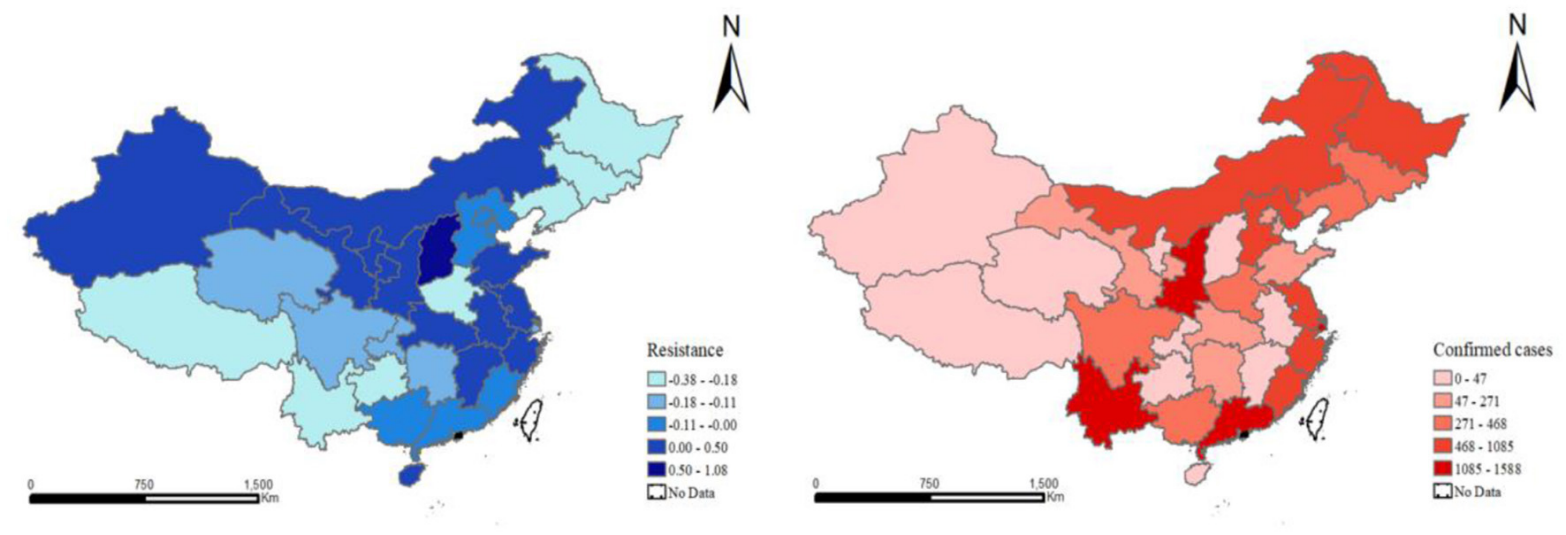
Regional economic resistance and geographic distribution of confirmed cases in provinces of China during COVID-19 in 2021.

According to Formulas (1) and (2), the economic resistance of provinces under COVID-19 outbreak in two periods was measured. When the resistance is 0.5, the regional impact of COVID-19 is <50% of the national level; when the resistance is −0.5, the regional impact of COVID-19 is more than 50% of the national level. The higher the resistance value, the stronger the regional economic resilience. Based on the dispersion of toughness values, the cross-section data of 2020 and 2021 were selected by natural discontinuity grading method and the spatial distribution maps of economic resistance and confirmed cases of 31 Provinces in China were drawn with ArcGIS software (Figures 1, 2). The images reveal significant spatial heterogeneity in COVID-19 resistance.

In 2020, the economic resistance of the western region is higher than the national average, followed by the northeast region and the central region. This means that the western and northeastern regions have been less affected by the pandemic, despite their structural economic disadvantage. Second, at the provincial level, more than one-third of the provinces have low resistance (less than zero) and are greatly affected by the epidemic. The distribution of confirmed COVID-19 cases is spatially consistent with regional economic resilience. Of course, provinces with more confirmed cases have been hit harder, with Hubei province being a prime example. In addition, the provinces with weak resistance showed obvious spatial agglomeration (both periods were consistent). The same is true for developed provinces and cities such as Beijing, Shanghai and Zhejiang, because they are located in coastal areas with dense transportation networks and a higher degree of economic openness, which makes them more vulnerable to COVID-19.

From the point of 2021, China's overall economic resistance obviously increased, the number of confirmed also declined obviously, among them with eastern and central parts of the most significant change. This means that in eastern and central regions to stabilize the outbreak earlier, the development trend of government to allocate limited resources, maximum reduce the negative impact on the economy the outbreak. It was also found that areas with more infected people were more affected by the epidemic and thus weakened their economic resistance, such as Henan Province, but the economic resistance of northeast China and some western regions decreased significantly with fewer infected people. In addition, the rapid and widespread spread of the virus is more likely due to the high urbanization rate and high population mobility in developed provinces and cities. So, in order to curb the spread of the epidemic, coastal provinces have restricted human activities and adopted a lifestyle of “working from home and taking online classes.” This has inevitably led to social and economic decline and weakened economic resistance, as confirmed by the distribution of confirmed cases.

The results from both periods also show that provinces with high numbers of infections had more negative effects on the economic resilience of their geographical proximity, partially offset by increased provincial economic resilience in 2021. In general, COVID-19 is not an industry-specific shock, but a global external crisis with predictable long-term impacts on human activities (51).

Given this, a region's economic system itself may play a limited role in influencing regional resilience. Consistent with part H1 of hypothesis, the more open and densely populated provinces have more exposure to novel coronavirus and less resistance to coronavirus.

## Determinants of the differential resistance to COVID-19

In order to determine the influencing factors of regional economic resistance under COVID-19, this paper classifies the selected independent variables based on ArcGIS Jenks optimal classification method, and takes the resistance index as the dependent variable. This study measured the regression coefficient between independent and dependent variables by Stata to indicate the direction of action between them. The results are shown in Table 2, where the absolute value of q value is in the range of [0,1]. The closer the q value of the variable is to 1, the more explanatory power it has to economic resilience. Statistics generally require a *p*-value. Based on the results of geographic detector, *p* < 0.1 is the criterion for judging significance, that is, the variable value is significant at 90% confidence level.

**Table 2:** Determinants of regional economic resilience.

**Factors**		**P**_D, U_	
		***q*** **-value**	***P*** **-value**
Economic strength	Economic openness (X1)	−0.165	0.525
	Real GDP per capita (X2)	0.379	0.093
Government power	Medical and health level (X3)	−0.336	0.011
	Grain and oil reserves (X4)	0.449	0.001
	Public safety (X5)	0.233	0.129
Social environment	Urbanization (X6)	0.373	0.000
	Transportation infrastructure (X7)	−0.387	0.015
Innovation ability	Science and technology level (X8)	0.510	0.002
	Average years of schooling (X9)	0.320	0.093

As can be seen from Table 2, economic level, medical and health level, grain and oil reserves, urbanization, transportation infrastructure, science and technology level, education level are the main factors influencing China's economic resistance to COVID-19. Among them, medical and health level (−0.336) and transportation infrastructure (−0.387) had a negative impact on regional resilience. The q values of these variables were all high and passed the significance test, indicating that these two variables played an important role in regional resistance to COVID-19. The q value of grain & oil reserves and science & technology level is the highest, so it has the greatest impact on regional economic resistance. The results further indicate that, first of all, global and regional trade in China's provinces has been negatively affected by the epidemic (25). Especially relying on international shipping and sea shipping and other ways of commodity trade suffered more serious damage, and showed a significant downturn. Although the q value is relatively small, this is consistent with the argument that COVID-19 has a more severe impact on globally connected cities (17). As a result, provinces with more open economies have been hit harder by the pandemic, showing weaker or even weaker resistance. Of course, this does not mean that regions with a high degree of openness are necessarily less resilient in the long term (referring to the subsequent recovery), but at least exhibit lower economic resilience during COVID-19 (17). Based on this, this can prove H1.

Secondly, it is found that medical & health level (−0.336), grain & oil reserves (0.449), and public safety (0.233) had significant effects on regional economic resistance under COVID-19. The q values of the three were all high, among which medical & health level and grain & oil reserves passed the significance test. It indicates that the government spending on grain & oil reserves and public safety can effectively resist the impact of COVID-19, which is based on the regional inheritance structure and the type of external shock. In order to contain the spread of the virus, the movement of people was restricted during the epidemic. Therefore, these two have played a direct and important role in ensuring people's basic living needs and responding to this public safety and health event. Everybody knows that a key aspect of containing the COVID-19 pandemic is the demand for and supply of medical supplies. As COVID-19 spread across Europe and North America, many countries quickly ran out of ppe, such as surgical masks and protective suits, due to transport disruptions and import and export trade restrictions that prevented them from sourcing these medical supplies from developing countries (24, 25). Since then, the world has witnessed a major shift in the global geography of medical supplies. China, in particular, has used its enormous mobilization to concentrate medical supplies where they are most needed – Wuhan, where the outbreak began, but it has not received the same attention in the surrounding areas of Hubei Province (24). The positive effect of such resource redistribution plan in China is more reflected in areas more severely affected by disasters. In most cases, such resource redistribution is a negative manifestation of “taking care of one and losing the other” (52). In Table 2, medical and health expenditure has a negative impact on regional resistance, with a large q value. This validates our claim that an economic variable clearly responding to the needs of the outbreak proved to be less regional resistant to the outbreak. It can be understood that the impact of the epidemic directly damages human life, and COVID-19 has a long latency time and can realize trans-temporal transmission (14). In case of confirmed cases or close contacts, immediate medical isolation is required, which is a huge demand for health care. The existing level of health care in the region is not sufficient to meet the impact of the outbreak and cannot meet the needs of all regions through a redistribution plan. Therefore, in order to protect human health and public safety, the local government had to sacrifice of economic benefits, timely adjust the structure of economic output, more revenue and manufacturing output for medical aid and so on, this inevitably makes economic stagnation, so as to produce a negative effect to economic resistance (24). Thus, it can be concluded that government forces can have a significant effect on regional resistance, but may have a negative effect. This is consistent with H2.

Thirdly, it is found that urbanization (0.373) and transport infrastructure (−0.387) have significant effects on regional economic resistance under COVID-19, both of which pass the significance test and have large q values, although transport infrastructure has a negative effect. In view of the role of social environment, it can be analyzed it from two aspects. On the one hand, due to the needs of national epidemic prevention and control, communities become the smallest unit of grid management, laying a solid foundation for effective epidemic prevention and control (53). This “grid governance” requires no one to leave their own community, and the person in charge of each grid buys and delivers food stores for the community collectively (18, 24). Grid management is initiated from urban community governance. Normal grid plays a positive role in rural governance, but its limitations are also obvious (54). Therefore, areas with a higher level of urbanization will be more likely to implement grid management and epidemic prevention and control measures during the COVID-19 outbreak, resulting in stronger regional resistance. On the other hand, the direct means to curb the spread of infectious diseases are the prohibition of movement of people and the interruption of transportation (55). Since the outbreak of COVID-19, emergency response and various traffic control measures have been launched across China to contain the spread of the epidemic (56). China's epidemic response has demonstrated that movement restrictions and traffic controls play an important role in regional economic recovery (17). Of course, the more complete the transport infrastructure, the more frequent the population movement, which is more likely to lead to the spread of the virus (21). Therefore, during the containment of the epidemic, movement restrictions and traffic control were implemented in the region, and the demand for medical supplies and daily necessities could not be met. As a result, transportation infrastructure had a negative effect on economic resistance. In other words, the social environment is not a favorable factor for COVID-19. It is not conducive to protecting the economy from the impact of COVID-19, leading to a weaker economic resistance. This does not mean that areas with a strong social infrastructure lack economic resilience in the long run, but it does, at least on the evidence available, reduce some resistance to COVID-19 in the short term. Therefore, the more perfect the social infrastructure environment, the more significant contribution to economic resistance, but not completely positive. This evidence is inconsistent with H3.

Finally, it is found that economic level (0.379), science and technology level (0.510) and education level (0.320) play a significant positive role in promoting regional economic resilience, passing the significance test and with large q values. This shows that regions with higher economic level are better able to resist external shocks. Considering that the higher the economic level of a region is, the more social capital that can be used for resistance and allocation after shocks is more conducive to the subsequent economic recovery (27). Regions with higher levels of science and technology and education have stronger regional innovation capacity, can quickly and effectively respond to external shocks and cooperate with national epidemic prevention and control policies, thus minimizing the spread of the epidemic and enhancing regional resistance (17, 24). It can be said that enhancing regional innovation capacity can enhance the region's ability to cope with the impact of COVID-19. This result will allow us to prove H4.

In addition, at the time of writing, the COVID-19 epidemic in China in 2022 showed a massive rebound, especially in Shanghai, the national economic center and financial center. The outbreak resulted in 600,000 infected people, and the whole city went into static management for more than 2 months. This rebound adds to our observations about the resilience of the region's economies—the COVID-19 pandemic is not going to end any time soon, it will take longer and more patience. Of course, the outbreak of the epidemic in Shanghai did not overturn our hypothesis and empirical results. Located in the coastal developed area, Shanghai is ahead of other provinces in all aspects, which is both an advantage and more infection risks under the impact of the COVID-19 epidemic. But this is consistent with the conclusion of the paper as a whole, although there are individual differences.

## Conclusion and enlightenment

The concept of regional economic resilience is considered to best explain and understand regional differences in response, adaptation and shock outcomes (7). Recent studies have highlighted the different impact of the type of shock itself on regional economic resilience (12). However, in the face of new external shocks, the research on this topic needs to be in-depth and rich, especially the lag effect of government forces and social environment in the economic system is often neglected. This article therefore focuses on how regional economies are responding to COVID-19—a serious global public health event and the greatest challenge humanity has faced since the 2008 financial crisis. This study includes (a) comparative analysis of economic resistance of various provinces in China, showing its spatial distribution and the evolution of confirmed cases; (b) the relationship between government power and the social environment and regional economic resilience was studied. The study used sample data from 31 Provinces in China, divided into four regions, specifically: northeast, central, eastern and western regions. The nine determinants of regional economic resilience can be divided into five categories: economic strength, government power, social environment and innovation capacity. Empirical evidence is the comparative difference in the economic resilience of different Chinese provinces in response to COVID-19 in 2020 and 2021. The findings show that the negative impact of COVID-19 varies from region to region and is determined by factors such as government power and social environment. There are three main conclusions in this paper: (1) from the regional perspective, the eastern and central regions are the first to be affected by the epidemic, and their economic resistance is lower than the national average. The western and northeastern regions are on the contrary, but the eastern and central regions can stabilize the development trend of the epidemic earlier; (2) from the perspective of provinces, developed provinces show stronger vulnerability (lower resistance) than backward provinces, and are more susceptible to the epidemic in the early stages of the impact; (3) government forces and social environment play an important role in regional economic resistance and adaptation in the initial stage of epidemic impact.

Conceptually, this paper contributes to the study of regional economic resilience in the face of COVID-19. Different from the 2008 financial crisis, the economic structure of the economy itself, such as industrial composition and industrial diversification, no longer plays a dominant role, but more depends on the inheritance of government power and social foundation. It is believed that COVID-19 is not directly to the economic crisis, it is first and foremost a health, social and public crisis of governance, to save human life and maintain the social and economic development for this (17). Therefore, only by comprehensively considering the role and contribution of various dimensions in the region can nations find a good treatment to resist the impact of COVID-19. First, more attention should be paid to region-specific policy lag effects, which provide dynamism and opportunities for economic resilience (57). Regional comparison factors, such as government strength and governance capacity related to national institutions (10). Regions that are doing well in the COVID-19 pandemic will serve as models for others to learn from, especially in terms of government strength. Second, the state and government play an irreplaceable role in shaping economic resistance through top-down scheduling and dictatorial ways of adjusting economic models based on regional economic and social foundations (18). For example, local governments can decide when to stagnate and revive the economy, depending on the type of external shock and their own economic benefits. Finally, China's redistribution plan is not suitable for large-scale and severe external shocks, and the original economic base and material reserves of the economic system need to be considered (24, 52). Of course, as the results presented in previous articles, these cases are based on short-term facing external impact area, the role of the government, however, in the long run, if the outbreak continue, unfortunately from national level the regional economic resilience to consider how to comprehensive utilization of multi-scale structural and environmental resources, in order to reduce the overall economic losses.

Finally, from the perspective of policy implications, China should take more active actions to cope with the dual challenges of internal and external shocks and the increasingly complex international economic situation at the critical moment of post-epidemic economic recovery. First, the COVID-19 response of individual Provinces in China depends on the path of action and the specific environment, and cannot be “one-size-fits-all.” Second, pay close attention to the key role of the government and the management of risk prevention, such as seeking international cooperation, allocating medical resources, taking restrictive measures, etc., need the timely response of the government, its ability to mobilize social and economic resources is irreplaceable. Third, prevention is always better than cure, and every region needs to support a financially sound and socially responsible public health policy and institutional mechanism to give people the best chance to escape devastating shocks such as the COVID-19 pandemic.

Over time, future studies can use time series data with more attributes to evaluate how the key findings in this study can be further empirically analyzed. Therefore, only over time, based on relevant data and empirical techniques can enable the study to establish an exact causal relationship, rather than the explanatory power evident in this study. At the same time, based on the research results, it is necessary to pay attention to the long-term economic recovery, consider the complete conceptual framework of economic resilience, and discuss the ability of regional economy to cope with external shocks from four aspects: vulnerability, resilience, robustness and resilience.

## Data availability statement

The original contributions presented in the study are included in the article/supplementary material, further inquiries can be directed to the corresponding author/s.

## Author contributions

TM: writing—original draft, methodology, and software. CT: writing—original draft. HZ: supervision, methodology, and writing—review and editing. CK: supervision, writing—review and editing, and funding acquisition. All authors contributed to the article and approved the submitted version.

## Conflict of interest

The authors declare that the research was conducted in the absence of any commercial or financial relationships that could be construed as a potential conflict of interest.

## Publisher's note

All claims expressed in this article are solely those of the authors and do not necessarily represent those of their affiliated organizations, or those of the publisher, the editors and the reviewers. Any product that may be evaluated in this article, or claim that may be made by its manufacturer, is not guaranteed or endorsed by the publisher.
